# Pharmacodynamic Evaluation and PK/PD-Based Dose Prediction of Tulathromycin: A Potential New Indication for *Streptococcus suis* Infection

**DOI:** 10.3389/fphar.2017.00684

**Published:** 2017-09-27

**Authors:** Yu-Feng Zhou, Hui-Min Peng, Ming-Xiao Bu, Ya-Hong Liu, Jian Sun, Xiao-Ping Liao

**Affiliations:** ^1^National Risk Assessment Laboratory for Antimicrobial Resistance of Animal Original Bacteria, South China Agricultural University, Guangzhou, China; ^2^Guangdong Provincial Key Laboratory of Veterinary Pharmaceutics Development and Safety Evaluation, College of Veterinary Medicine, South China Agricultural University, Guangzhou, China

**Keywords:** tulathromycin, new indication, *Streptococcus suis*, PK/PD modeling, dose prediction

## Abstract

Tulathromycin is the first member of the triamilide antimicrobial drugs that has been registered in more than 30 countries. The goal of this study is to provide a potential new indication of tulathromycin for *Streptococcus suis* infections. We investigated the pharmacokinetic and *ex vivo* pharmacodynamics of tulathromycin against experimental *S. suis* infection in piglets. Tulathromycin demonstrated a relatively long elimination half-life (74.1 h) and a mean residence time of 97.6 h after a single intramuscular administration. The minimal inhibitory concentration (MIC) and bactericidal concentration in serum were markedly lower than those in broth culture, with Mueller–Hinton broth/serum ratios of 40.3 and 11.4, respectively. The post-antibiotic effects were at 1.27 h (1× MIC) and 2.03 h (4× MIC) and the post-antibiotic sub-MIC effect values ranged from 2.47 to 3.10 h. The ratio of the area under the concentration–time curve divided by the MIC (AUC/MIC) correlated well with the *ex vivo* antimicrobial effectiveness of tulathromycin (*R*^2^ = 0.9711). The calculated AUC_12h_/MIC ratios in serum required to produce the net bacterial stasis, 1-log_10_ and 2-log_10_ killing activities were 9.62, 18.9, and 32.7, respectively. Based on the results of Monte Carlo simulation, a dosage regimen of 3.56 mg/kg tulathromycin was estimated to be effective, achieving for a bacteriostatic activity against *S. suis* infection over 5 days period. Tulathromycin may become a potential option for the treatment of *S. suis* infections.

## Introduction

*Streptococcus suis* is one of the most important pathogens in the swine industry causing septicemia, meningitis, septic arthritis, and pneumonia, leading to large economic losses worldwide (Feng et al., 2014; Goyette-Desjardins et al., 2014). In addition, *S. suis* is an emerging zoonotic agent responsible for the severe systemic infections with high morbidity and mortality rates in humans, especially in people who are in close occupational contact with pigs and pork products (Gurung et al., 2015). Over the past decades, the number of reported cases of *S. suis* infections has significantly increased in humans, with two large outbreaks in humans in 1998 and 2005 in China (Li et al., 2010). More importantly, a concise solution for streptococcosis on pig farms is not available and the effective antimicrobials are still needed to control *S. suis* infections (Van Hout et al., 2016). In general, penicillin is used to treat and control infections due to *S. suis*. However, penicillin-resistant strains and strains highly resistant to other β-lactam antibiotics have been reported (Lun et al., 2007). Therefore, novel antibiotics or effective treatment strategies against *S. suis* infections are urgently needed.

Tulathromycin is a long acting, semi-synthetic macrolide antibiotic, developed for the treatment and control of bacterial respiratory diseases in cattle and swine (El Badawy et al., 2014). The list of approved indications by the US Food and Drug Administration (FDA) and European Medicines Agency (EMA) includes *Mannheimia haemolytica, Actinobacillus pleuropneumoniae*, and *Pasteurella multocida* (Evans, 2005). Additionally, tulathromycin is also registered for the treatment of interdigital necrobacillosis caused by *Porphyromonas levii* (Villarino et al., 2013). Of note, label indications may vary between countries and may be regionally updated (Villarino et al., 2013). However, tulathromycin is currently not indicated for *S. suis* infections. Given that tulathromycin has activity against a wide range of respiratory associated pathogens and other macrolides are used for treatment of *Streptococcus pneumoniae*, we wanted to further investigate its *ex vivo* activity of tulathromycin against *S. suis* infections. We especially wanted to include *S. suis* capsular serotype 2 strains because these are the most virulent and dominant pathogenic serotypes in severe infections in both swine and humans globally (Lun et al., 2007; Feng et al., 2014). Moreover, we studied the effect of varying testing matrices on susceptibility, the post-antibiotic effect (PAE) and post-antibiotic sub-MIC (minimal inhibitory concentration) effect (PA-SME) of tulathromycin against *S. suis*. This finding could provide a framework for setting the basis for new treatment strategies and for the discovery of new macrolides with further applications in veterinary and human medicine (Villarino et al., 2013).

## Materials and Methods

### Animals and Experimental Design

Eight healthy castrated crossbred piglets (Duroc × Landrace × Yorkshire; four males, four females) weighing 20.5–23.6 kg were used for a two-period crossover study. The piglets were allowed *ad libitum* access to water and antibiotic-free food. They were maintained in accordance with National Standards for Laboratory Animals of China (GB 14925-2010). The animal experiment procedures were approved by the Animal Research Committees of South China Agricultural University. Each piglet received tulathromycin (Draxxin Pfizer Animal Health, Sandwich, Kent, United Kingdom) at a single dose of 2.5 mg/kg by intravenous (IV) and intramuscular (IM) injections.

### Sampling Procedures and Analysis of Tulathromycin

Blood samples (5 mL volume) were collected by venipuncture of the anterior vena cava into vacutainers (Becton, Dickinson and Company, Oxford, Oxon, United Kingdom) lacking anticoagulant, and stored in the dark. All samples were centrifuged at 3000 × *g* for 10 min at 4°C, and the supernatants stored at -80°C prior to assay for drug concentration and for measurement of *ex vivo* antimicrobial activity.

All frozen serum samples were thawed at room temperature. Roxithromycin (0.20 μg/mL) was added as internal standard (Wang et al., 2012). For drug extraction, 0.5 mL sample aliquots were transferred to centrifuge tubes, and then mixed with 0.5 mL of acetonitrile. After vortexing (1 min) and centrifugation (12,000 × *g*, 10 min), the supernatant was filtered through a 0.22 μm nylon syringe filter. The drug levels in serum were determined by a liquid chromatography-tandem mass spectrometry method (Agilent 1200 HPLC system; API 4000 triple quadrupole mass spectrometer) as previously reported (Wang et al., 2012). The limit of quantification and detection were 0.01 and 0.005 μg/mL, respectively. The correlation coefficient (*R*) was above 0.999 in the linear range of 0.01–0.5 μg/mL. All serum samples that had concentrations above 0.5 μg/mL were diluted proportionally with control serum prior to extraction with acetonitrile. The recoveries of tulathromycin were >85%, and relative standard deviations (SDs) for both interday and intraday were <10% at all tested concentrations. In addition, the protein binding of tulathromycin was determined at spiked concentrations of 0.05, 0.5, and 5 μg/mL using ultrafiltration methods as our previously reported (Zhou et al., 2015).

### Pharmacokinetic Analysis

Tulathromycin time–concentration data in serum of individual piglets were analyzed using WinNonlin non-compartmental modeling (Version 6.1, Pharsight, St. Louis, MO, United States). The maximal drug concentration (*C*_max_) was determined directly from the data with *T*_max_ defined as the time of the first occurrence of *C*_max_. The linear trapezoidal rule was used to calculate area under the concentration–time curve (AUC). Additional PK parameters such as terminal half-life (T_1/2_λ_z_), total body clearance (Cl), volume of distribution at steady state (*V*_ss_), and mean residence time (MRT) were also determined. The absolute bioavailability (*F*) of tulathromycin was calculated according to the standard equations (Zhou et al., 2015): *F*% = AUC_IM_/AUC_IV_ × 100%. All PK parameters are presented as mean ± SD.

### *In Vitro* Susceptibility Testing

Sixteen strains of *S. suis* isolated from swine suffering septicemia and pneumonia were used in this study. Routine determinations of MIC and minimal bactericidal concentration (MBC) of tulathromycin against these isolates were conducted using a broth microdilution technique in accordance with CLSI methods (CLSI, 2008). The standard solution of tulathromycin was prepared in 0.0015 M citrate buffer, with pH adjusted to 7.0 with 0.015 M citric acid or 0.02 M NaOH. For determination of MICs in Mueller–Hinton broth (MHB), calf serum collected commercially from animals <2 years old was supplemented at 5% (v/v) as required. MIC was defined as the lowest drug concentration of tulathromycin that inhibited the visible bacterial growth in medium (Zhou et al., 2016). MBCs in MHB and serum were determined using the spot-plate technique to obtain a 3-log_10_ reduction in bacterial count relative to the initial inoculum (Illambas et al., 2013). In addition, to determine the influence of test matrix on MICs, the tulathromycin MIC for *S. suis* ATCC 43765 was further determined in MHB, 100% serum and varying proportions of serum (25, 50, and 75%) mixed with MHB. All experiments were performed three times on separate occasions to ensure reproducibility.

### Antimicrobial Activities of Tulathromycin and PAEs

*In vitro* time–kill curves of tulathromycin against *S. suis* ATCC 43765 were established using the broth clinical method as previously described (Lees et al., 2016). Briefly, tulathromycin concentrations ranging from 0.25 to 16× MIC for *S. suis* were prepared in pre-warmed MHB. For control growth curves, MHB lacking drug was used. The bacteria were diluted in MHB to 10^6^ CFU/mL and then incubated in an orbital shaking incubator at 37°C for 24 h. Aliquots of 50 μL of each culture were sampled and the viable counts (CFU/mL) were determined by 10-fold serial dilutions and spot-plate counts after incubation for 0, 3, 6, 9, and 12 h. The lowest detectable count was 40 CFU/mL. Each concentration was performed in triplicate to ensure reproducibility.

*Ex vivo* killing curves were determined as per above using the serum samples obtained from piglets at all time points from 0 to 360 h after IM dosing with an initial inoculum of 10^6^ CFU/mL of *S. suis*. Serum sample were pre-filtered through a 0.22 μm membrane to clear any possible bacterial contamination (Zhou et al., 2016).

In addition, the PAEs and PA-SMEs of tulathromycin were performed by a combination of spectrophotometric method and viable count method as our previously reported (Zhou et al., 2016). The optical density was converted into the amount of organisms based upon the preliminary standard curve (Supplementary Figure S1). The PAE and PA-SME were calculated as follows (Spivey, 1992; Zhou et al., 2016): PAE = *T* -*C*; PA-SME = *T*_PA_ -*C*, where *T* and *T*_PA_ is the time required for viable counts of bacteria to increase by 1-log_10_ CFU in drug removal phase and sub-MIC-treated phase, respectively; *C* is the time for untreated control.

### PK/PD Modeling and Data Analysis

Based on *in vitro* MIC data and *in vivo* PK parameters, surrogate markers of antibacterial efficacy; i.e., the ACU_12h_/MIC over 12 h, was calculated for each tulathromycin concentration using the *ex vivo* killing curves. PK/PD modeling was used to describe the relationship between *ex vivo* AUC_12h_/MIC in serum and the antimicrobial effect measured as changes in log_10_ CFU after 12 h incubation. This model was established using the sigmoid *E*_max_ model that derived from the Hill equation (Toutain and Lees, 2004; Zhou et al., 2016): *E* = *E*_0_ + (*E*_max_ -*E*_0_) × *C*_e_*^N^*/(EC_50_*^N^* + *C*_e_*^N^*), where *E*_0_ is the log_10_ change in visible counts of drug-free control, *E*_max_ is the maximal antibacterial effect, EC_50_ is the PK/PD index target producing a 50% of maximal effect, *C*_e_ is the PK/PD index (AUC_12h_/MIC), and *N* is the Hill coefficient that represented the slope of dose–response curve. All data were analyzed using the non-linear WinNonlin regression program (Version 6.1, Pharsight). The coefficients of determination (*R*^2^) were used to estimate the variance associated with regression modeling with the PK/PD index. In addition, AUC_12h_/MIC targets corresponding to the net bacterial stasis, 1-log_10_ and 2-log_10_ reductions in bacterial counts after 12 h incubation were calculated by the *E*_max_ model for *S. suis* infection in serum.

### Dose Regimen Prediction

Although tulathromycin is currently not indicated for *S. suis* infections, the approved dose regimen (2.5 mg/kg) for *M. haemolytica* and *P. multocida* seems not to be adequate to cover the overall MIC variabilities. In order to deduce a more rational dose regimen potentially for the treatment of *S. suis* infections, a 10,000-subject Monte Carlo simulation (Oracle Crystal Ball; Oracle Corporation, Redwood Shores, CA, United States) was employed to predict the dose distributions as follows (Toutain et al., 2002, 2016):

$$\text{Dose}\;(\text{for}\;\text{5}\;\text{days}\;\text{of}\;\text{activity})\;\text{=}\;\frac{{\text{Cl}}_{\text{for}\;\text{5}\;\text{days}}\;\times \;\text{SF}\;\times \;{\text{MIC}}_{\text{distribution}}}{fu\;\times \;F}$$

Where the dose regimen is the given amount to produce a specific antibacterial activity over 5 days; Cl is the body clearance (L/kg/5 day); SF is a scaling factor, obtained by dividing the PK/PD index target, in this case, AUC_12h_/MIC, by 12 h, resulting in a SF of 0.8 for a bacteriostatic activity. *F* is the bioavailability, and *fu* is the free drug fraction.

The MIC distribution was obtained from our own laboratory in combination with data from some previous studies (El Garch et al., 2016; Sweeney et al., 2017). In general, only susceptible *S. sui*s would be successfully treated with tulathromycin and should therefore be considered to calculate a potential dose regimen. Owing to no CLSI breakpoint available for tulathromycin against *S. suis*, we selected all the MICs equal to or less than 64 μg/mL, which was the recently reported MIC_90_ value of tulathromycin for *S. suis* isolated from pigs in the United States and Canada 2011–2015 (Sweeney et al., 2017).

## Results

### Pharmacokinetics of Tulathromycin in Piglets

**FIGURE 1:**
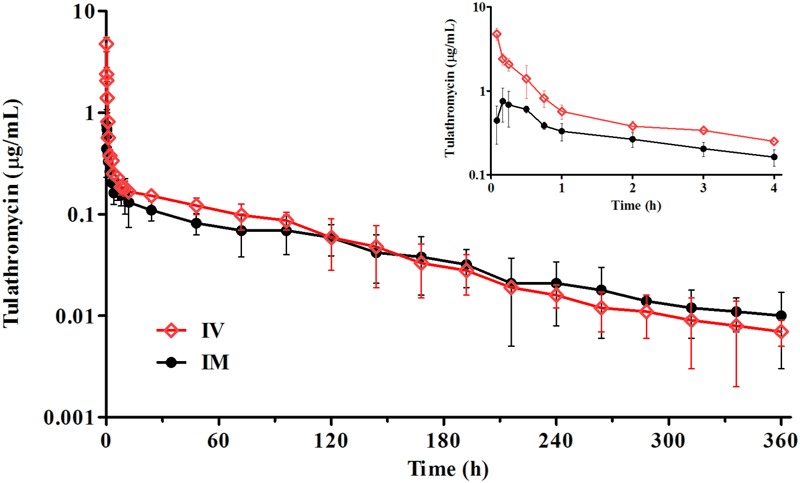
The time–concentration profiles of tulathromycin in porcine serum following a single IV or IM administration of 2.5 mg/kg (*n* = 8).

After a single IM dosing, the peak concentration (*C*_max_) of tulathromycin (0.82 ± 0.25 μg/mL) was reached at 0.23 ± 0.14 h. The drug concentrations of tulathromycin in serum exceeded 0.10 μg/mL up to 48 h and were still detectable (mean; 0.01 μg/mL) at 360 h (**Figure 1**). The IM bioavailability of tulathromycin was 78.3 ± 19.2%, and the serum terminal half-life (*T*_1/2_λ_z_) and MRT after IV dosing (2.5 mg/kg) were 69.4 and 89.3 h, respectively. These were fairly long and resulted from both a large volume of distribution (*V*_ss_; 11.3 L/kg) and a low body clearance (Cl; 115 mL/kg/h) (**Table 1**).

**Table 1:** Pharmacokinetic parameters of tulathromycin in porcine serum after single intravenous (IV) and intramuscular (IM) administrations at 2.5 mg/kg.^a^

Parameter^b^	Unit	IV dosing	IM dosing
*T*_max_	h	0.08 ± 0.01	0.23 ± 0.14
*C*_max_	μg/mL	4.78 ± 0.54	0.82 ± 0.25
λ_z_	1/h	0.01 ± 0.01	0.01 ± 0.01
*T*_1/2_λ_z_	h	69.4 ± 5.71	74.1 ± 10.6
AUC_last_	μg⋅h/mL	21.8 ± 3.54	19.1 ± 4.18
AUC_infinity_	μg⋅h/mL	22.5 ± 3.79	20.9 ± 4.46
Cl	mL/kg/h	115 ± 19.6	–
Cl/*F*	mL/kg/h	–	126 ± 11.5
*V*_ss_	L/kg	11.3 ± 2.72	–
MRT	h	89.3 ± 10.9	97.6 ± 11.3
*F*	%	–	78.3 ± 19.2

*^a^Values are means ± standard deviation (SD); (*n* = 8). ^b^*T*_*max*_, time following dosing at which the maximal concentration (*C*_*max*_) occurred; *T_*1/2*λ*z*_*, terminal half-life; λ_*z*_, first-order rate constant associated with the terminal portion of the curve; AUC_*last*_, area under the concentration–time curve from 0 to the last sampling time; AUC_*infinity*_, area under the concentration–time curve from 0 to infinity; Cl, body clearance; Cl/*F*: clearance scaled by bioavailability; *V*_*ss*_, volume of distribution at steady state; MRT, mean residence time; *F*, bioavailability.*

The protein binding of tulathromycin in porcine serum ranged from 32.3 to 39.1% at spiked levels of 0.05, 0.5, and 5 μg/mL, with a mean binding rate of 36.3%. The results indicated that the free/unbound fraction of tulathromycin in serum would be approximately 63.7%.

### MICs and MBCs of Tulathromycin for *S. suis* Isolates

The test matrix influenced the susceptibility of tulathromycin for *S. suis* isolates. The MIC and MBC values determined in MHB were significantly higher than those determined in porcine serum, with MHB/serum ratios of 40.3 and 11.4, respectively (**Table 2**). The MBC/MIC ratios ranged from 2.46 to 8.75 for both test matrixes. For all the porcine *S. suis* isolates used for this study, the tulathromycin MICs in serum were in the range of 0.007–0.5 μg/mL, with MIC_50_ and MIC_90_ values of 0.06 and 0.25 μg/mL, respectively.

**Table 2:** Tulathromycin MIC and MBC values (μg/mL) and summary of test medium effect on MICs and MBCs for *S. suis* isolates (*n* = 16).^a^

Test matrix	MIC	MBC	MBC/MIC ratio
MHB	2.14 (0.57)	5.28 (1.10)	2.46
Serum	0.05 (0.04)	0.46 (0.12)	8.75
MHB/Serum ratio^b^	40.3	11.4	–

*^a^The MIC and MBC values represent as geometric mean (SD) of 16 *S. suis* isolates. ^b^Comparison of MHB/Serum ratio differences: *P* < 0.01.*

To further compare the matrix effects, the tulathromycin MIC for *S. suis* ATCC 43765 was determined in porcine serum and in MHB supplemented to contain 25, 50, and 75% serum. The 25% serum incorporated in MHB reduced MICs more than threefold compared to that determined in 100% MHB. When increasing supplementation with serum further to 50 and 75%, an overall decrease in MIC values for *S. suis* ATCC 43765 of at least 10-folds was observed, resulting in MICs as low as 0.04–0.08 μg/mL compared to a consistent MIC of 1 μg/mL under the standard condition (**Table 3**). A visible concentration-dependent decrease in MICs was observed with increasing proportions of serum mixed with MHB.

**Table 3:** Tulathromycin MICs (mg/L) for *S. suis* ATCC 43765 in MHB and 25, 50, 75, and 100% porcine serum.^a,b^

Matrix	MHB	Serum percentage in MHB^c^			Serum
		25%	50%	75%	
Geometric mean	1.00	0.31	0.08	0.04	0.03
SD	0.00	0.12	0.03	0.01	0.02

*^a^The MIC values are the geometric mean of three independent replicates. ^b^*S. suis* ATCC 43765 is an reference strain of capsular serotype 2. ^c^Significantly different from MIC determined in MHB (*P* < 0.01).*

### Antimicrobial Activities of Tulathromycin and PAEs

**FIGURE 2:**
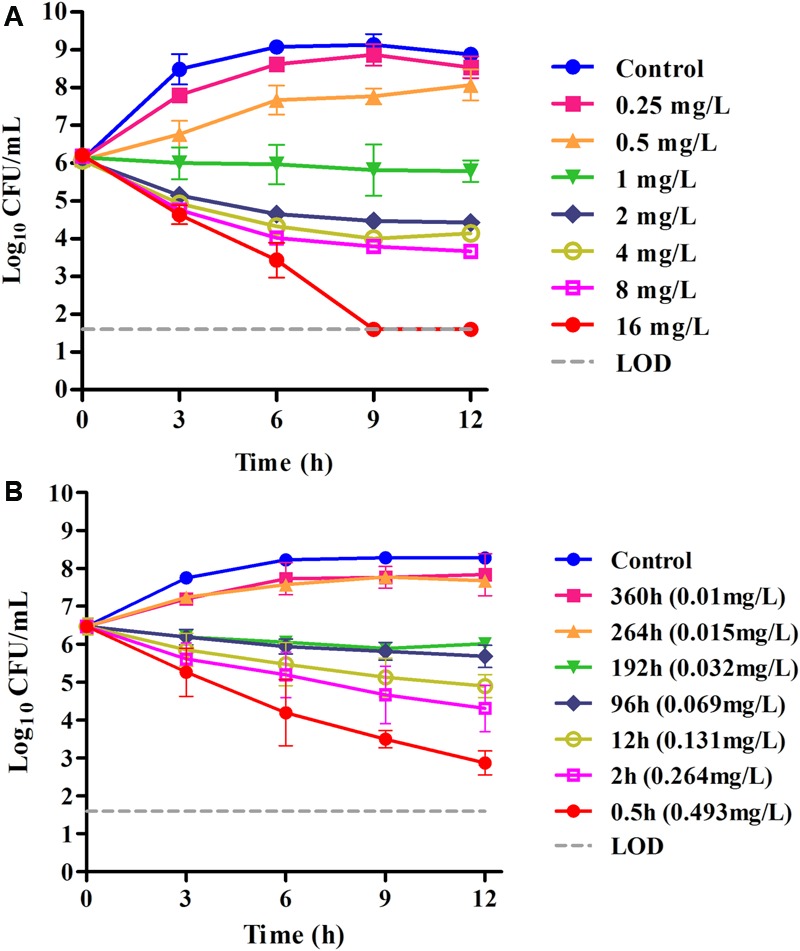
**(A)** *In vitro* time–kill curves of tulathromycin against *S. suis* ATCC 43765 in the MHB medium (MIC_MHB_ = 1 μg/mL); **(B)** *ex vivo* inhibition of bacterial growth in porcine serum before and after IM administration of tulathromycin (MIC_serum_ = 0.031 μg/mL). Numerical values on the right for each panel are tulathromycin concentrations in serum.

We performed *in vitro* time–kill curves of tulathromycin in the range of 0.25–16 multiples of MIC against *S. suis.* Tulathromycin at 0.25 and 0.5× MIC produced only a slight inhibition of bacterial growth compared to control. However, persistent growth inhibition was observed when the bacteria were exposed to 1 μg/mL tulathromycin. At 2–8× MIC, the higher drug concentrations could not lead to a more rapid and greater reduction, indicating a time-dependent killing pattern for *S. suis*. Additionally, the bacterial counts were reduced by roughly 4-log_10_ CFU/mL after 9 h exposure to tulathromycin at 16× MIC (**Figure 2A**).

In serum collected up to 192 h, tulathromycin exerted effective growth inhibition or bactericidal effects after 12 h of incubation. However, no bactericidal activity was obtained for serum collected at 264 or 360 h, and these were followed by a 1.2-log_10_ CFU regrowth. Despite the lower MIC achieved in serum, there was a somewhat slower rate of killing in serum compared with the same multiples of MIC in MHB. Even for serum collected at 0.5 h (about 16× MIC_serum_), a reduction in bacterial counts to the detection limit was not achieved after 12 h of incubation (**Figure 2B**).

**FIGURE 3:**
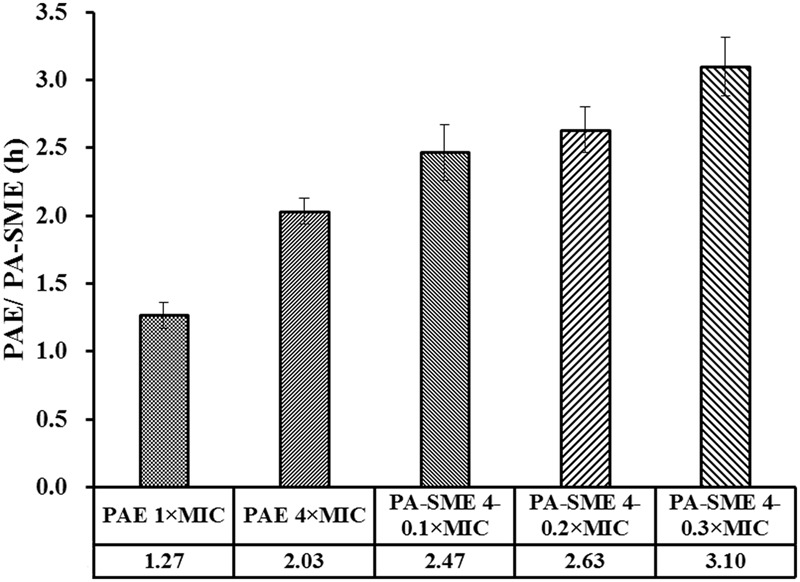
PAE and PA-SME values (h) of tulathromycin against *S. suis* ATCC 43765. The PA-SMEs were determined after initial exposure to tulathromycin at 4× MIC. The error bars represent SD (*n* = 3).

Removal of the tulathromycin after drug exposure for 1 h resulted in delayed regrowth (PAE) that ranged from 1.27 and 2.03 h (**Figure 3**). However, a longer persistent suppression of bacterial regrowth for *S. suis* was observed during the sub-MIC-treated phase (Supplementary Figure S2), with PA-SMEs of 2.47–3.10 h.

### PK/PD Analysis and Dose Regimen Prediction

**FIGURE 4:**
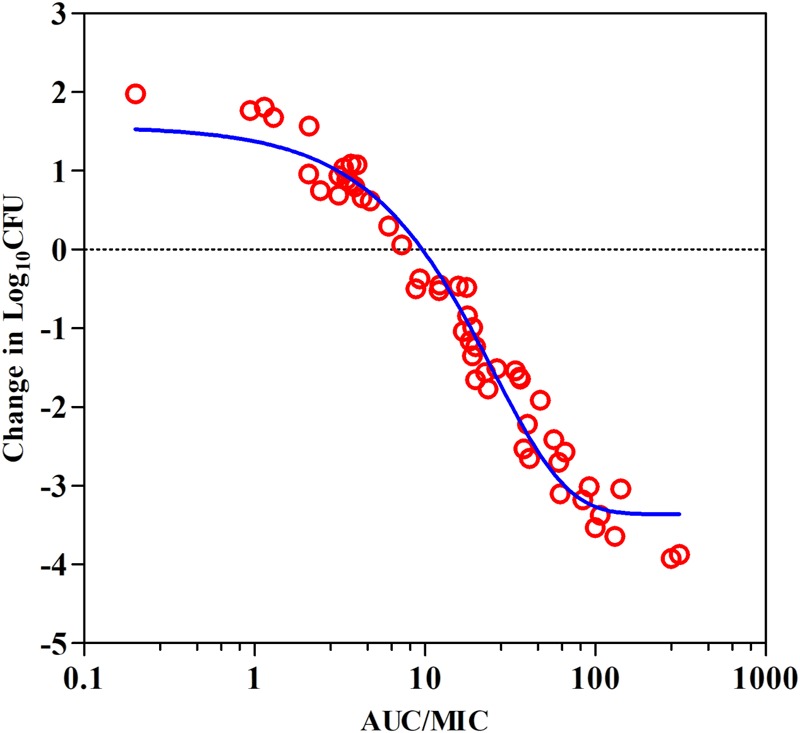
Plots of *ex vivo* AUC/MIC ratios versus the change of bacterial counts between 0 and 12 h (log_10_ CFU/mL) against *S. suis* ATCC 43765 in the porcine serum. The line represents the curve of predicted values based on the sigmoid *E*_max_ equation, and the points represent serum samples collected at time points from 0 to 360 h.

Data derived from PK/PD modeling of the *ex vivo* antibacterial activities showed a steep concentration–effect curve with the maximal bactericidal effects (*E*_max_) of 3.9-log_10_ CFU reduction in bacterial counts (**Figure 4**). The calculated AUC_12h_/MIC values in serum required to produce the net bacterial stasis, 1-log_10_ and 2-log_10_ killing activities were 9.62, 18.9, and 32.7, respectively (**Table 4**).

**Table 4:** PK/PD analysis of data acquired from *ex vivo* time–kill studies of tulathromycin against *S. suis* in porcine serum.

Parameter (units)^a^	Values
*E*_0_ (log CFU/mL)	1.76
*E*_max_ (log CFU/mL)	-3.87
*E*_max_ -*E*_0_ (log CFU/mL)	-5.63
EC_50_ (h)	17.9
Slope (*N*)	1.18
AUC_12h_/MIC for static effect (h)	9.62
AUC_12h_/MIC for 1-log_10_ kill effect (h)	18.9
AUC_12h_/MIC for 2-log_10_ kill effect (h)	32.7

*^a^*E*_*0*_, difference in number of bacteria counts (log_*10*_ CFU/mL) in drug-free sample between 0 and 12 h; *E*_*max*_, difference in greatest amount of bacterial reduction (log_*10*_ CFU/mL); EC_*50*_ is the AUC/MIC value producing 50% of the maximal antibacterial effect; *N*, the slope of AUC/MIC response curve.*

**FIGURE 5:**
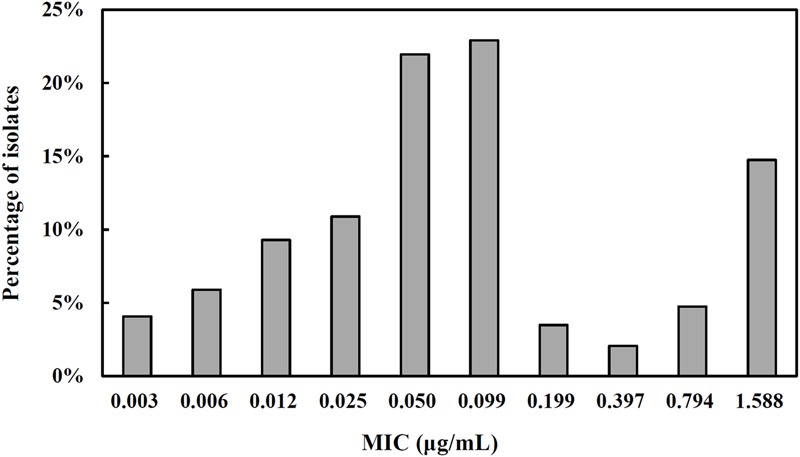
The MIC distribution for tulathromycin against *S. suis* (*n* = 441). The isolates were obtained from our own laboratory in combination with data from some previous studies (El Garch et al., 2016; Sweeney et al., 2017). All reported MIC in broth matrix was transformed into equivalent MICs in serum by a scaling factor of 40.3 to take into account the serum effect.

**FIGURE 6:**
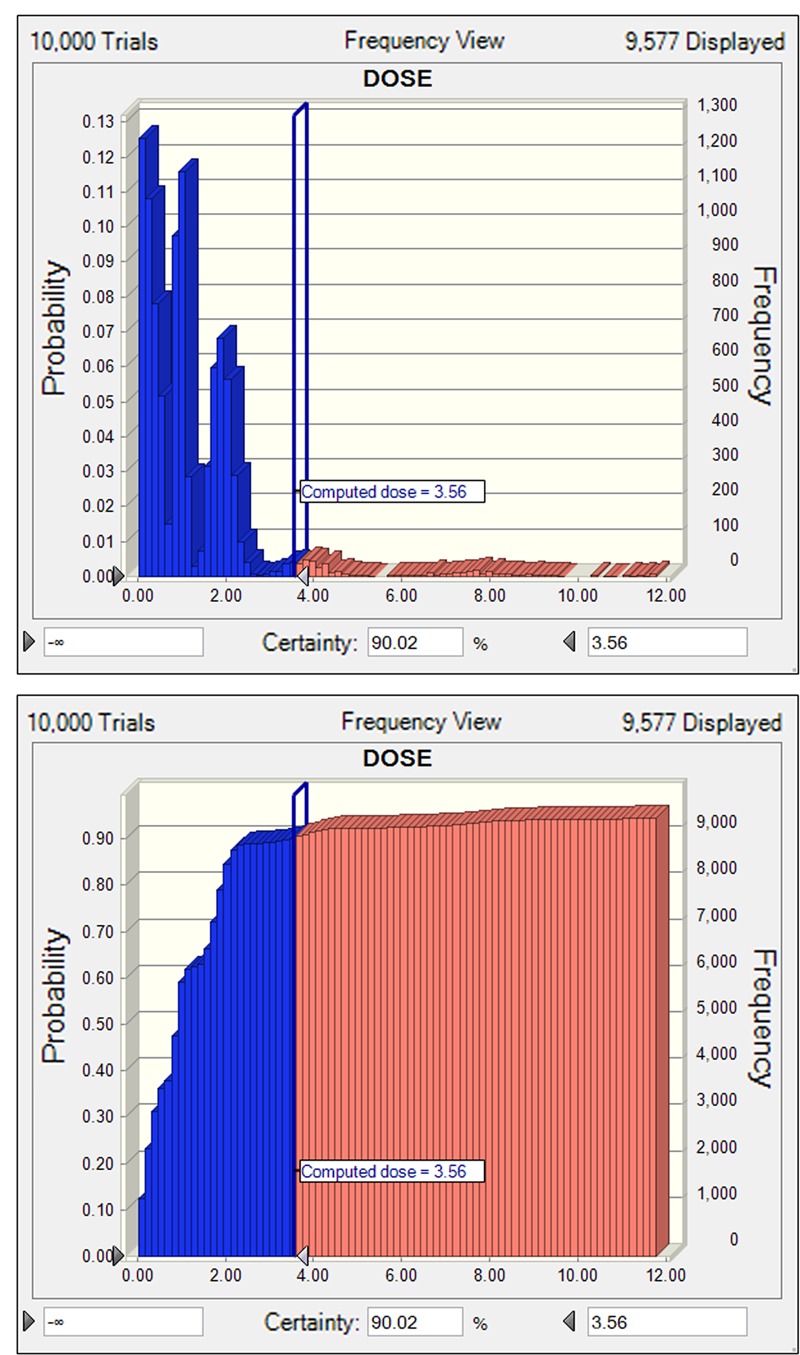
The non-cumulative **(A)** and cumulative **(B)** distributions of tulathromycin doses for *S. suis*, as predicted by a PK/PD model for a duration of 5 days activity in terms of probability of target attainment (PTA). The vertical bar indicates the computed target dose at the cumulative PTA of 90%. Dose (0–12 mg/kg) is indicated on the *x*-axis.

Tulathromycin should be administered as a single dose able to cover at least 5 days activity that required a mean Cl/*F* of 15.1 L/kg. After intramuscular dosing of tulathromycin in piglets, the *ex vivo* AUC_12h_/MIC target associated with net bacterial stasis was 9.62 h. This is equivalent to consider that a bacteriostatic activity can be obtained with a serum drug concentration equal to 0.8-folds the MIC. The distribution of tulathromycin MIC in broth matrix was transformed into vector equivalent MICs in serum by a scaling factor of 40.3 to take into account the serum effect (**Figure 5**). The unbound fraction is of 63.7. Accordingly, based on the results of Monte Carlo simulation, for a cumulative probability of target attainment of 90%, the predicted dose was 3.56 mg/kg (**Figure 6**), which was estimated to be effective to achieve a bacteriostatic activity against *S. suis* isolates over 5 days period.

## Discussion

Tulathromycin is an effective veterinary-specific antibiotic for the treatment of swine and cattle respiratory diseases caused by *A. pleuropneumoniae* and *P. multocida*, with the MIC_90_ values of 16 and 2 μg/mL, respectively (Benchaoui et al., 2004; Villarino et al., 2014). Although previous studies have demonstrated the AUC/MIC ratio was the predictive PK/PD index that best correlated with the antimicrobial activity of tulathromycin (Evans, 2005; Villarino et al., 2013), there is a paucity of pharmacodynamic studies of tulathromycin against *S. suis* to provide a comparison to the current studies. To our knowledge, this is the first study designed to evaluate the *ex vivo* antimicrobial activity and PK/PD relationships of tulathromycin against *S. suis*. In this study, tulathromycin demonstrated time-dependent bactericidal activity against *S. suis*. After a 2.5 mg/kg IM administration, tulathromycin is characterized by a rapid rate of absorption and large systemic availability, with a high absolute bioavailability of 78.3%. Of note, in general, the local inflammation caused by infections may significantly interfere with the kinetics of drug distribution and protein binding in plasma (Dello et al., 1988; Gajda et al., 2016). Likewise, the prior pharmacokinetics study of tulathromycin in pigs with pulmonary inflammation revealed markedly longer elimination half-lives in plasma and in lung (126 and 165 h, respectively) (Gajda et al., 2016). However, rapidly distribution and long persistence of tulathromycin in lung tissues do not indicated a direct relationship of the effective drug concentrations associated with located pathogens (Villarino et al., 2014). The pharmacokinetics of free tulathromycin in pulmonary and bronchial epithelial lining fluids should be further evaluated.

The clinical efficacy of tulathromycin may be underestimated by the *in vitro* MIC measurements. Consistent with other macrolides, tulathromycin MICs measured in a biologic matrix (serum) was markedly lower than that determined in the artificial matrix (defined broth medium) (Barry and Fuchs, 1991; Godinho, 2008). This finding is supported by a similar result from a previous study using calf serum in which the presence of 50% serum during the MIC test decreased MICs by 12-fold for *M. haemolytica* and ninefold for *P. multocida* (Lees et al., 2016). For *S. suis* isolates, our results demonstrated that incremental supplementation of culture matrix with serum resulted in noticeable shifts to lower MICs by at least 5-log_2_ dilution steps. This increased susceptibility response in porcine serum was analogous to that produced by tildipirosin, a novel macrolide, which produced progressive reductions in MICs with increasing amounts of serum for six bacterial species harvested from pigs (Rose et al., 2013). Additionally, an early antibacterial activity study in human serum demonstrated that the killing rate of erythromycin against *Escherichia coli* and *Staphylococcus aureus* was increased in the presence of serum (Pruul and McDonald, 1992).

In general, macrolides poorly diffuse across the outer membrane of most Gram-negative bacteria (Nikaido, 1998; Buyck et al., 2012). The mechanism for markedly increased susceptibility of *S. suis* to tulathromycin in porcine serum is probably related to the downregulation of *opr*M and increased permeability of bacterial outer-membrane when grown in biological fluids (Pruul and McDonald, 1992; Buyck et al., 2012). Consistent with this view, the previous *in vitro* susceptibility study of clarithromycin showed a considerable enhancement of antibacterial activity against *P. aeruginosa* in RPMI 1640 medium and other eukaryotic cell culture media as compared with the conventionally used broths (Buyck et al., 2012). More recently, the similar negative clinical consequence was observed when testing activity of macrolide and ketolide was assessed in CA-MHB, suggesting that measuring MICs in RPMI-1640 could be easily implemented to phenotypically detect acquired resistance to macrolides in *P. aeruginosa* from cystic fibrosis patients (Mustafa et al., 2017). In this case, serum MIC is more predictive to provide a reasonable surrogate for studies of *in vivo* antimicrobial activity when the objective is to establish a rational dosing regimen.

Tulathromycin is the first member of a novel subclass of macrolides known as triamilides, which can be positively charged at the appropriate pH (Villarino et al., 2013). The antimicrobial activity of tulathromycin may be in theory related to the matrix pH. Consistently, results from previous studies determining the effect of pH on MICs indicated greater potency of tulathromycin (lower MIC) against *M. haemolytica* and *P. multocida* as pH increased in the range of 7.0–8.2 (two extremes of pH in extracellular fluids under physiological conditions) (Lees et al., 2016). Therefore, matrix pH seems likely to be another important factor accounting for markedly augmented activity in porcine serum compared with MHB (Dalhoff et al., 2005; Lees et al., 2016). In addition, as reported for other macrolides, tulathromycin may tend to be accumulated within lysosomes due to the relatively higher pKa (>8.6) and acidic environment of lysosomes (Villarino et al., 2013). However, given that the MICs of some pathogens are inversely related to pH, the intracellular activity of tulathromycin is probably limited (Tulkens, 1991; Villarino et al., 2013).

Similar with other macrolides, tulathromycin is considered a bacteriostatic agent against *S. aureus* and *E. coli* (Villarino et al., 2013). However, in this study, an *in vitro* bactericidal activity was noted at four- or eightfolds the MIC for *S. suis*. The further PK/PD-based modeling suggested that animal dose regimens should supply AUC_12h_/MIC of tulathromycin at least 9.62 to achieve a net bacteriostatic activity against *S. suis*. Based on the free drug AUC_12h_ of 5.86 μg⋅h/mL in piglets, it is apparent that the current approved dose (2.5 mg/kg) could not achieve such PD target for *S. suis* infection with a MIC_serum_ of ≥0.6 μg/mL (i.e., MIC_MHB_ of 24 μg/mL). Therefore, tulathromycin, if used for the treatment of *S. suis* infection, would benefit from larger doses than are commonly used in clinical practice.

## Conclusion

The present study investigated for the first time the *ex vivo* antimicrobial activities and PK/PD relationships of tulathromycin against *S. suis* in piglet serum. Based on the PK data and MIC distribution, we predicted the dosage distribution of tulathromycin, and estimated an appropriate dose (3.56 mg/kg) for a bacteriostatic activity against *S. suis* infection over 5 days period. The marked differences in MIC values between artificial media and serum highlight the importance of matrix effects on susceptibility testing. Additionally, our findings suggested that PD evaluation data for tulathromycin should be derived in biological fluids such as serum for preparing the dosage regimens for clinical therapeutic use.

## Author Contributions

X-PL conceived of this study and participated in its design and coordination. Y-FZ and H-MP designed the experiment and drafted the manuscript. Y-FZ, H-MP, and M-XB carried out the animal experiments and time–kill curve studies. Y-HL and JS participated in the data analysis and revision of manuscript. All authors read and approved the final manuscript.

## Conflict of Interest Statement

The authors declare that the research was conducted in the absence of any commercial or financial relationships that could be construed as a potential conflict of interest.
