# Supplementary material for: Pharmacodynamic Evaluation and PK/PD-Based Dose Prediction of Tulathromycin: A Potential New Indication for Streptococcus suis Infection
Source: Front Pharmacol. 2017 Sep 27;8:684. doi: 10.3389/fphar.2017.00684 (PMC5627010; doi:10.3389/fphar.2017.00684)
Supplement: Supplementary file 1 [file Image_1.PDF]

Supplementary Material

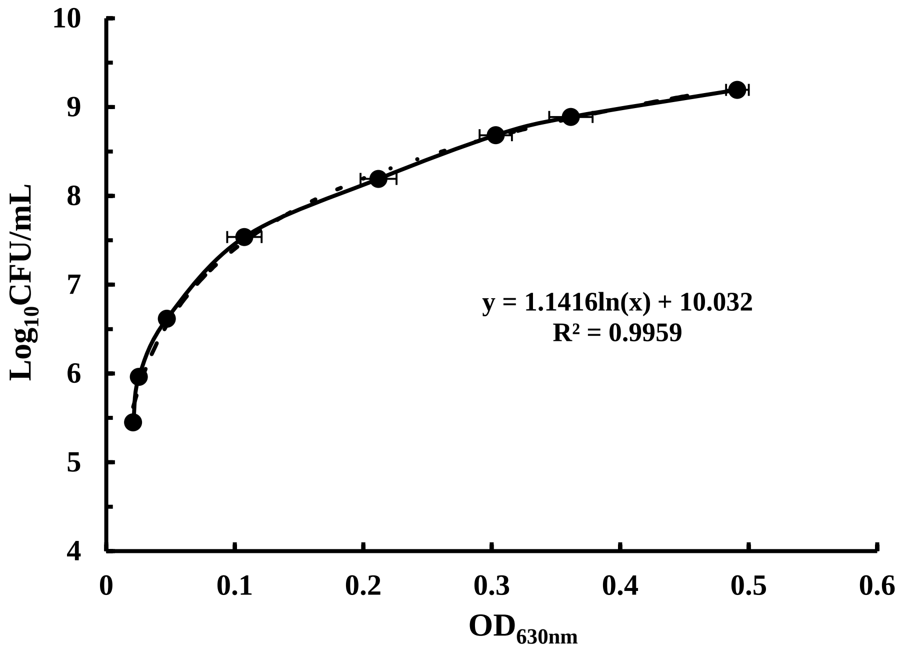

Figure S1. Standard curve was constructed by regression of the viable count and optical density (OD_{630nm}) value of the inoculum. The solid line represents the curve of observed value, and the dash line is predicted value. The equation was fitted as follows: $y = 1.1416 \ln(x) + 10.032$, the coefficient of determination (R^2) was 0.996.

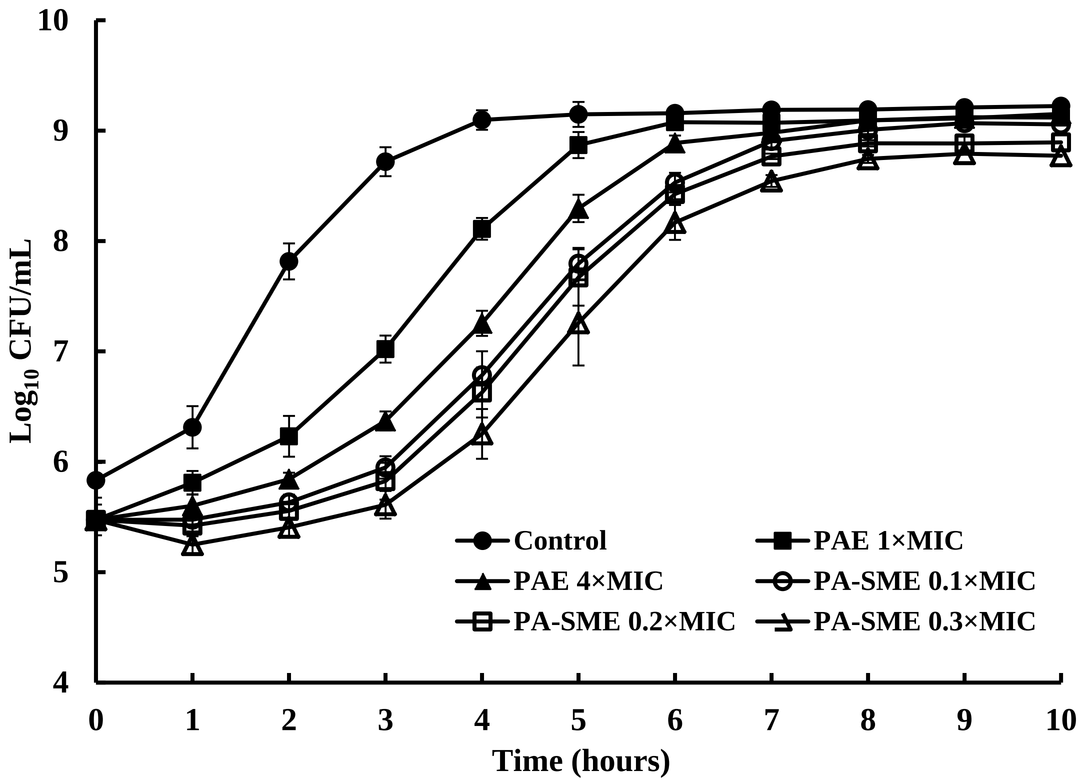

Figure S2. Regrowth curves of *S. suis* ATCC 43765 exposed to different levels of tulathromycin. The PA-SMEs were determined after initial exposure to tulathromycin at 4×MIC (MIC_{MHB} = 1 µg/mL).
